# Correction to “L1CAM‐associated extracellular vesicles: A systematic review of nomenclature, sources, separation, and characterization”

**DOI:** 10.1002/jex2.95

**Published:** 2023-06-21

Gomes, D. E., & Witwer, K. W. (2022). L1CAM‐associated extracellular vesicles: A systematic review of nomenclature, sources, separation, and characterization. *Journal of Extracellular Biology*, 1, e35. https://doi.org/10.1002/jex2.35

Tables 1, 2, 3, 4, 5 in the article were formatted incorrectly so that the references cited in each table could not be identified in conjunction with the article's reference list. The correct tables are included below.

**TABLE 1:** Nomenclature in EV L1CAM studies

**Term**	**Number**	**References**
EV	20	Athauda et al.; 2019; Bhargava et al., 2021; Cha et al., 2019; Chawla et al.; 2019; Cressati et al., 2021; Dagur et al., 2020; Eitan et al., 2017; Fu et al., 2020; Gomes et al., 2015; Kodidela et al., 2020; Kumar et al., 2021; Mansur et al., 2021; Nasca et al., 2021; Norman et al., 2021; Pulliam et al., 2020; Suire et al., 2017; Winston et al.,2016; Zhao et al., 2020; Zou et al., 2020
Exosome	32	Anastasi et al., 2021; Athauda et al., 2019; Dagur et al., 2020; Fauré et al., 2006; Goetzl et al., 2020; Gu et al., 2020; Gutwein et al., 2005; Herrero et al., 2019; Jiang et al., 2020; Jiang et al., 2021; Keller et al., 2009; Kodidela et al., 2020; Mullins et al., 2017; Nogueras‐Ortiz et al., 2020; Pace et al., 2019; Patterson et al., 2018; Peltz et al., 2020; Rani et al., 2019; Shi et al., 2014; Shi et al., 2016; Si et al., 2019; Stoeck et al., 2006; Sun et al., 2017; Sun et al., 2019; Trnka et al., 2012; Walker et al., 2021; Webber et al., 2014; Winston et al., 2019; Yang et al., 2009; Yang et al., 2011; Yuyama et al., 2019
Other	4	Gutwein et al., 2003; Keller et al., 2009; Shi et al., 2016; Stoeck et al., 2006

**TABLE 2:** Sources of EVs in L1CAM EV studies

**Source**	**n (of 51)**	**References**
Blood products (plasma, serum)	40	
Serum	10	Athauda et al., 2019; Chawla et al., 2019; Cressatti et al., 2021; Dagur et al., 2020; Jiang et al., 2020; Jiang et al., 2021; Keller et al., 2009; Si et al., 2019; Walker et al., 2021; Yuyama et al., 2019
Plasma	31	Anastasi et al., 2021; Bhargava et al/, 2021; Cha et al., 2019; Cressatti et al., 2021; Eitan et al., 2017; Goetzl et al., 2015; Goetzl et al., 2019; Goetzl et al., 2020; Gu et al., 2020; Herrero et al., 2019; Jiang et al., 2020; Kodidela et al., 2020; Kumar et al. 2021; Mansur et al., 2021; Mullins et al., 2017; Nasca et al., 2021; Nogueras‐Ortiz et al., 2020; Norman et al., 2021; Patterson et al., 2018; Peltz et al., 2020; Pulliam et al., 2020; Shi et al., 2014; Shi et al., 2016; Suire et al., 2017; Sun et al., 2018; Sun et al., 2019; Walker et al., 2021; Winston et al., 2016; Winston et al., 2018; Winston et al., 2019; Zhao et al., 2020
*Blood + Other*	*10*	
Cell culture‐conditioned medium	14	Anastasi et al., 2021; Dagur et al., 2020; Faure et al., 2006; Gomes et al., 2015; Gutwein et al., 2003; Herrero et al., 2021; Keller et al., 2009; Nogueras‐Ortiz et al., 2020; Pace et al., 2019; Stoeck et al., 2006; Webber et al., 2014; Winston et al., 2019; Yang et al., 2009; Yang et al., 2011
Brain tissue	2	Dagur et al., 2020; Yuyama et al., 2019
CSF	2	Cressatti et al., 2021; Norman et al., 2021
Urine	2	Cressatti et al., 2021; Trnka et al., 2012
Ascites	2	Gutwein et al., 2005; Kellar et al., 2009
Saliva	2	Cressatti et al., 2021; Rani et al., 2019

**TABLE 3:** Separation and concentration methods

**Separation/concentration method**	**n (of 51)**	**References**
Affinity (immunoprecipitation/capture, FACS)	35	Anastasi et al., 2021; Athauda et al., 2019; Bhargava et al., 2021; Cha et al., 2019; Chawla et al., 2019; Cressatti et al., 2021; Dagur et al., 2020; Eitan et al., 2017; Fu et al., 2020; Goetzl et al., 2015; Goetzl et al., 2019; Goetzl et al., 2020; Gu et al., 2020; Jiang et al., 2020; Jiang et al., 2021; Kumar et al., 2021; Mansur et al., 2021; Mullins et al., 2017; Nasca et al., 2021; Nogueras‐Ortiz et al., 2020; Norman et a., 2021; Patterson et al., 2018; Peltz et al., 2020; Pulliam et al., 2020; Shi et al., 2014; Si et al., 2019; Suire et al., 2017; Sun et al., 2018; Sun et al., 2019; Winston et al., 2016; Winston et al., 2018; Winston et al., 2019; Zhao et al., 2020; Zou et al., 2020
PEG or PEG‐based kit	30 (26 combining PEG and IP)	Altevogt et al., 2020; Alvarez‐Erviti et al., 2011; Angiolini et al., 2019; Athauda et al., 2019; Bhargava et al., 2021; Campbell et al., 2021; Cha et al., 2019; Chawla et al., 2019; Cocucci et al., 2015; Coleman et al., 2015; Colombo et al., 2015; Cressatti et al., 2021; Dagur et al., 2020; Dickens et al., 2017; Eitan et al., 2017; Faissner et al.; 1984; Fogel et al., 2003; Gavert et al., 2008; Goetzl et al., 2015; Goetzl et al., 2019; Goetzl et al., 2020; Gu et al., 2020; Gutwein et al., 2000; Gutwein et al., 2003; Gutwein et al., 2005; Harding et al., 1983; Herrero et al., 2019; Hill, 2019; Jiang et al., 2020; Johnstone, 1991; Kodidela et al., 2020; Kumar et al., 2021; Li et al., 2010; Linneberg et al., 2019; Lötvall et al., 2014; Maness et al., 2007; Mansur et al., 2021; Manu et al., 2021; Mateescu et al., 2017; Meldolesi, 2021; Mullins et al., 2017; Nasca et al., 2021; Nogueras‐Ortiz et al., 2020; Norman et al., 2021; Patterson et al., 2018; Peltz et al., 2020; Pulliam et al., 2019; Pulliam et al., 2020; Rani et al., 2019; Russell et al., 2019; Samatov et al.; 2016; Shankar et al.; 2017; Si et al., 2019; Sugawa et al., 1997; Suire et al., 2017; Sun et al., 2017; Sun et al., 2019; Théry et al., 2018; Thompson et al., 2016; Upadhya et al., 2021; Vandendriessche et al., 2020; Vassileff et al., 2020; Verweij et al., 2021; Walker et al., 2021; Winston et al., 2016; Winston et al., 2018; Winston et al., 2019; Witwer et al., 2019; Witwer et al., 2021; Yang et al., 2009; Yuyama et al., 2019; Zhao et al., 2020; Zou et al., 2020
Differential ultracentrifugation	17	Dagur et al., 2020; Faure et al., 2006; Gomes et al., 2015; Gutwein et al., 2003; Gutwein et al., 2005; Herrero et al., 2019; Keller et al., 2009; Norman et al., 2021; Pace et al., 2019; Patterson et al., 2018; Shi et al., 2016; Stoeck et al., 2006; Trnka et al., 2012; Webber et al., 2014; Yang et al., 2009; yang et al., 2011; Yuyama et al., 2019
Density gradient or cushion ultracentrifugation	9	Dagur et al., 2020; Faure et al., 2006; Gutwein et al., 2003; Gutwein et al., 2005; Keller et al., 2009; Norman et al., 2021; Stoeck et al., 2006; Webber et al., 2014; Yuyama et al., 2019
Size exclusion chromatography	3	Anastasi et al., 2021; Herrero et al., 2019; Norman et al., 2021

Distribution of concentration/separation methods in n=51 publications. Several studies used multiple workflows. Some combined more than one method in at least one workflow (n=39), and these were counted in all respective categories. FACS=fluorescence‐activated cell sorting; PEG=polyethylene glycol. Ultrafiltration of various types was also an occasional component of EV workflows (not shown).

**TABLE 4:** Anti‐L1CAM antibodies used for reported capture of EVs

**L1CAM antibody clone**	**n (of 35)**	**Epitope**	**Example references**
5G3	19	Ectodomain, immunoglobulin‐like domains 1 to 2; near N‐terminus	Anastasi et al., 2021; Chawla et al., 2019; Cressati et al., 2021; Eitan et al., 2017; Goetzl et al., 2015; Goetzl et al., 2019; Goetzl et al., 2020; Gu et al., 2020; Kumar et al., 2021; Mansur et al., 2021; Mullins et al., 2017; Nasca et al., 2021; Nogueras‐Ortiz et al., 2020; Patterson et al., 2018; Suire et al., 2017; Sun et al., 2017; Walker et al., 2021; Winston et al., 2018; Winston et al., 2019
EPR23241‐224	1	Similar epitope to 5G3	Norman et al., 2021
UJ127 (UJ127.11)	7	Ectodomain, fibronectin type III repeats	Anastasi et al., 2021; Fu et al., 2020; Jiang et al., 2020; Jiang et al., 2021; Shi et al., 2014; Shi et al., 2016; Si et al., 2019
C2C (or 2C2)	1	Intracellular, C‐terminus; cross‐reactive with CHL1	Zou et al., 2020
LS‐C470565	1	Intracellular, C‐terminus	Dagur et al., 2020
Unspecified	7	N/A	Athauda et al., Bhargava et al., 2021; Cha et al., 2019; Peltz et al., 2020; Pulliam et al., 2020; Sun et al., 2019; Zhao et al., 2020

**TABLE 5:** Use of physical characterization techniques

**Technique**	**n (of 51)**	**References**
Optical/Electrical Methods (OEM): RPS, NTA, DLS	22	Anastasi et al., 2021; Bhargava et al., 2021; Chawla et al., 2019; Cressatti et al., 2021; Dagur et al., 2020; Eitan et al., 2017; Gomes et al., 2015; Gu et al., 2020; Herrero et al., 2019; Kodidela et al., 2020; Kumar et al., 2021; Mansur et al., 2021; Nogueras‐Ortiz et al., 2020; Pulliam et al., 2020; Rani et al., 2019; Shi et al., 2016; Sun et al., 2017; Sun et al., 2019; Webber et al., 2014; Winston et al., 2016; Zhao et al., 2020; Zou et al., 2020
Non‐Optical Microscopy (NOM): TEM, SEM, AFM	19	Anastasi et al., 2021; Dagur et al., 2020; Faure et al., 2006; Fu et al., 2021; Gutwein et al., 2003; Herrero et al., 2019; Jiang et al., 2020; Kodidela et al., 2020; Nogueras‐Ortiz et al., 2020; Norman et al., 2021; Pace et al., 2019; Shi et al., 2014; Shi et al., 2016; Si et al., 2019; Winston et al., 2016; Yang et al., 2011; Yuyama et al., 2019; Zhao et al., 2020; Zou et al., 2020
Multiple	10	Anastasi et al., 2021; Dagur et al., 2020; Fu et al., 2021; Gomes et al., 2015; Herrero et al., 2019; Kodidela et al., 2020; Nogueras‐Ortiz et al., 2020; Shi et al., 2016; Winston et al., 2016; Zhao et al., 2020; Zou et al., 2020
None (in several cases, techniques were mentioned but results not reported)	19	Athauda et al., 2019; Cha et al., 2019; Goetzle et al., 2019; Goetzl et al., 2015; Goetzle et al., 2020; Gutwein et al., 2005; Jiang et al., 2021; keller et al., 2009; Mullins et al., 2017; Nasca et al., 2021; Patterson et al., 2018; Peltz et al., 2020; Stoeck et al., 2006; Suire et al., 2017; Trnka et al., 2012; Walker et al., 2021; Winston et al., 2018; Winston et al., 2019; Yang et al., 2009

We apologize for this error.

